# Transcriptional and Physiological Changes during *Mycobacterium tuberculosis* Reactivation from Non-replicating Persistence

**DOI:** 10.3389/fmicb.2016.01346

**Published:** 2016-08-31

**Authors:** Peicheng Du, Charles D. Sohaskey, Lanbo Shi

**Affiliations:** ^1^Office of Advanced Research Computing, Rutgers, The State University of New JerseyNew Brunswick, NJ, USA; ^2^VA Long Beach Healthcare System, United States Department of Veterans AffairsLong Beach, CA, USA; ^3^Public Health Research Institute, New Jersey Medical School, Rutgers, The State University of New JerseyNewark, NJ, USA

**Keywords:** tuberculosis, gene expression profiling, RNA-Seq, reactivation, lag phase, metabolism and physiology, transcription regulon/subnetwork

## Abstract

*Mycobacterium tuberculosis* can persist for years in the hostile environment of the host in a non-replicating or slowly replicating state. While active disease predominantly results from reactivation of a latent infection, the molecular mechanisms of *M. tuberculosis* reactivation are still poorly understood. We characterized the physiology and global transcriptomic profiles of *M. tuberculosis* during reactivation from hypoxia-induced non-replicating persistence. We found that *M. tuberculosis* reactivation upon reaeration was associated with a lag phase, in which the recovery of cellular physiological and metabolic functions preceded the resumption of cell replication. Enrichment analysis of the transcriptomic dynamics revealed changes to many metabolic pathways and transcription regulons/subnetworks that orchestrated the metabolic and physiological transformation in preparation for cell division. In particular, we found that *M. tuberculosis* reaeration lag phase is associated with down-regulation of persistence-associated regulons/subnetworks, including DosR, MprA, SigH, SigE, and ClgR, as well as metabolic pathways including those involved in the uptake of lipids and their catabolism. More importantly, we identified a number of up-regulated transcription regulons and metabolic pathways, including those involved in metal transport and remobilization, second messenger-mediated responses, DNA repair and recombination, and synthesis of major cell wall components. We also found that inactivation of the major alternative sigma factors SigE or SigH disrupted exit from persistence, underscoring the importance of the global transcriptional reprogramming during *M. tuberculosis* reactivation. Our observations suggest that *M. tuberculosis* lag phase is associated with a global gene expression reprogramming that defines the initiation of a reactivation process.

## Introduction

Tuberculosis, caused by the intracellular pathogen *Mycobacterium tuberculosis*, became the top deadly infectious disease, responsible for the death of about 1.5 million people in 2014 (WHO, 2015). Tuberculosis manifests in several clinical stages. In the majority of individuals the initial infection transitions into an asymptomatic latent state where *M. tuberculosis* is believed to exist within host granulomas in a non-replicating persistent state with low metabolic activity (Hobby et al., 1954; Wayne and Sohaskey, 2001; Dutta and Karakousis, 2014). This special physiological state of *M. tuberculosis* accounts for the successful evasion from the host immunity as well as the long duration of treatment regimen with anti-tuberculosis drugs, which leads to increased cases of toxicity and low rates of treatment completion (Hobby et al., 1954; Dutta and Karakousis, 2014; Muñoz et al., 2015; Turetz and Ma, 2016). Hostile environmental factors responsible for the establishment of this physiological state in granulomas include low oxygen, poor nutrition, low pH, and both oxidative and nitrosative stresses generated by host immune response (Dutta and Karakousis, 2014). However, when the host immune response is compromised, such as by coinfection with HIV, *M. tuberculosis* can resume growth, leading to disease activation (Lawn et al., 2002). Recent advances also reveal that *M. tuberculosis* has evolved the ability to exacerbate pathophysiological progression in granulomas, leading to disease progression and eventual dissemination of the bacteria (Ramakrishnan, 2012). Given the fact that the major cause of morbidity and mortality from tuberculosis results predominantly from reactivation of an undiagnosed latent infection (WHO, 2015), a better understanding of reactivation biology may yield not only novel assays that can identify persons at highest risk for progression to clinical disease, but also novel therapeutic strategies to shorten the treatment of latent *M. tuberculosis* infection.

Various mechanisms of *M. tuberculosis* adaptation to hostile environments have been elucidated using *in vitro* and *in vivo* models. One key aspect is the activation of bacterial transcription regulatory networks (TRN) including two component systems, alternative sigma factors, and transcription factors, which couple environmental stress signals to changes in gene expression, leading to changes in bacterial metabolism and physiology (Zahrt and Deretic, 2001; Malhotra et al., 2004; Manganelli et al., 2004b; He et al., 2006; Leistikow et al., 2010; Flentie et al., 2016). For example, the MprA-SigE signaling subnetwork has been regarded as one of the key adaptation subnetworks during bacterial entry to persistent state (Manganelli et al., 2001; Talaat et al., 2004; He and Zahrt, 2005; He et al., 2006). Another well-characterized *M. tuberculosis* stress response system is the ~48 gene persistence-associated DosR regulon, which is induced in response to stress environments such as decreased oxygen tension and nitric oxide (Sherman et al., 2001; Voskuil et al., 2003; Leistikow et al., 2010). Using multiple models, we characterized several key bacterial metabolic and physiological adaptations during *M. tuberculosis* transition to non-replicating persistence, which included induction of DosR genes (Shi et al., 2003), a shift of respiratory pathways from energy efficient aerobic respiration to less energy efficient pathways (Shi et al., 2005), and rerouting carbon flux from the generation of energy and biosynthetic precursors during bacterial growth to the synthesis of storage compound triacylglycerol in non-replicating bacteria (Shi et al., 2010). However, in contrast to our improved understanding on mechanisms of *M. tuberculosis* persistence, very little is known about the molecular mechanism of its reactivation from persistence, especially during early stages of the reactivation. An early study characterized the *M. tuberculosis* reactivation response using cultures after a short-term hypoxia treatment (Sherrid et al., 2010), which may not fully reveal changes that occur during reactivation of a latent infection in humans. Studies with fully anaerobic dormant cultures have shown that both the DosR regulator and alanine dehydrogenase (*ald)* facilitate an optimal recovery upon reaeration (Leistikow et al., 2010; Giffin et al., 2016).

To understand the molecular events leading to bacterial reactivation, we characterized physiological changes and transcriptomic dynamics by RNA-Seq of *M. tuberculosis* during reactivation after extended hypoxia treatment. Our data indicate that prior to the resumption of *M. tuberculosis* cell division, a global gene expression reprogramming with characteristics of regrowth initiation orchestrates the recovery of bacterial metabolic and physiological functions. Our findings also suggest that *M. tuberculosis* reactivation is a programmed exit from persistence, which requires a coordinated change of gene expression in various regulatory and metabolic pathways.

## Materials and methods

### Bacterial culture, wayne low oxygen model, and regrowth cultures

The *M. tuberculosis sigH* and *sigE* mutants and the complemented strains from H37Rv background were kindly provided by Dr. Maria Gennaro lab. They were originally generated by the lab of Dr. Issar Smith at the Public Health Research Institute, New Jersey Medical School, Rutgers, The State University of New Jersey (Manganelli et al., 2001, 2002). Cultures of *M. tuberculosis* were grown in Dubos Tween Albumin (DTA) medium [0.05% (w/v) tryptone, 15 mM asparagine, 153 μM Tween-80, 7 mM KH_2_PO_4_, 18 mM Na_2_HPO_4_, 189 μM ferric ammonium citrate, 83 μM MgSO_4_, 5 μM CaCl_2_, 348 nM ZnSO_4_, 400 nM CuSO_4_, 15 mM NaCl, 0.5% bovine serum albumin fraction V and 42 mM dextrose] (Becton Dickinson), and subjected to the Wayne low oxygen model with a headspace ratio of 0.5 (Wayne and Hayes, 1996). At day 25, after ~16 days of anaerobiosis, cultures were opened, mixed with an equal volume of fresh DTA medium and vortexed to introduce oxygen into the system. Seven milliliter of regrowth culture was distributed into 25 ml tubes and incubated at 37°C under aerobic condition with magnetic bar stirring at 450 rpm (Datta et al., 2011). Regrowth dynamics were monitored by measuring the optical density (OD) at 580 nm and samples were harvested at defined time points for the measurement of physiological parameters including bacterial colony forming units (CFU), cellular oxygen consumption, ATP levels, or for RNA-Seq. For survival study tubes were opened at intervals for CFU enumeration by plating and then subsequently discarded.

### ATP measurements

One milliliter of regrowth culture was harvested by centrifugation, and the pellet was snap-frozen in dry-ice/ethanol for subsequent ATP extraction and measurement, as described (Wayne and Hayes, 1996). Briefly, cell pellets were resuspended in 1 ml of HEPES buffer (0.025 M, pH 7.75 and 0.03% Tween-80). One hundred microliter of cell suspension was mixed with 40 μl of chloroform, and the resulting sample was heated at 80°C for 20 min, followed by the addition of 4.9 ml of HEPES buffer. One hundred microliter of this ATP extract was mixed with an equal volume of luciferase assay reagent derived by mixing luciferase assay buffer and the lyophilized luciferase assay substrate (Luciferase Assay Systems, Promega Corporation). Light output was recorded in a luminometer (Glomax-R, Promega Corporation). Light units were converted to ATP concentrations using a standard curve of light units obtained with a series of ATP standard dilutions (Sigma-Alrich). ATP content was normalized to bacterial CFUs.

### Oxygen consumption by methylene blue oxidation

Twenty-five-day hypoxic cultures were mixed with methylene blue to a final concentration 0.0003% and oxygen was reintroduced into the system by vortexing. The resulting mixture was transferred into small tubes to full capacity and tightly sealed. Decolorization of culture-methylene blue mix was monitored by measurement of absorbance at 665 nm, as described (Sohaskey, 2005).

### Total RNA extraction

For RNA extraction, regrowth cultures were first treated with RNAprotect Bacteria Agents (Qiagen) for 15 min to stabilize RNA according to manufacturer's recommendations and harvested by centrifugation. Cell pellets were kept at −80°C for subsequent total RNA extraction, as described (Shi et al., 2015).

### RNA-seq

90 bp pair-end sequencing using Illumina platform with HiSeq 2000 System (TruSeq SBS KIT-HS V3, Illumina) was carried out by BGI Americas. Briefly, rRNAs were first removed from total RNAs using the Ribo-Zero™ Magnetic Gold Kit (Epicenter); after mRNA fragmentation, first strand cDNA synthesis was carried out using Super Script II (Invitrogen) reverse transcription system; after the second strand cDNA synthesis, the double strand cDNAs were repaired and their 3′ end adenylated for adapter ligation with RNA index adaptors, followed by Uracil-N-glycosylase digestion; the dsDNA library was enriched by PCR using PCR primer cocktail and master mix; the amplified library was validated by measurement of broad size distribution using Agilent 2100 Bioanalyzer and quantification by QPCR using TaqMan probes; the qualified library (250 ~ 300 bp) was amplified on cBot to generate cluster on the flowcell (TruSeq PE Cluster Kit V3–cBot–HS, Illumina); and finally the amplified flowcell was sequenced pair-end on the HiSeq 2000 System (TruSeq SBS KIT-HS V3, Illumina). The raw image files were processed by on-instrument real time analysis (RTA v1.18.64) to generate per cycle BCL basecall files as primary sequencing output, and Illumina Off-Line Basecaller (v1.9.4) combines these per-cycle BCL files from a run and translates them into QSEQ files. Raw reads were trimmed of adaptor sequences, sequencing primers, and multiplex barcode using SOAPnuke software (BGI), and clean reads of 90 bp were obtained by filtering out low quality reads including those with unknown nucleotides larger than 5%, reads with more than 50% of the bases' quality less than 10, and short reads with adaptors. About 2 GB clean data (corresponding to ~10 million reads), which were ~90% of the total reads, were obtained per sample.

### Bioinformatics and statistical analysis

RNA-Seq reads from triplicate samples at each time point were mapped to the *M. tuberculosis* H37Rv genome with BOWTIE2 (Langmead and Salzberg, 2012), and processed with R package DESeq (Anders and Huber, 2010). Greater than 96% of reads mapped properly paired to *M. tuberculosis* genome for each sample. Expression of each individual gene was obtained in each sample by counting the number of reads mapped to the gene and normalizing the counts with DESeq. Fold change and *p*-values were also calculated by DESeq. Expression profiles of all the genes were deposited in NCBI Gene Expression Omnibus (GEO) (GEO# GSE83814). To identify significantly changed metabolic pathways and groups of genes that function in closely related categories, we performed the Fisher's Exact Test for genes with fold changes ≥1.5 against KEGG pathway and KEGG BRITE hierarchies (http://www.genome.jp/kegg/), respectively. Additionally, to identify the significantly changed transcription regulons/subnetworks, we first assembled a more inclusive *M. tuberculosis* transcriptional regulatory network (TRN) by complementing existing networks with interactions from literatures (He et al., 2006; Balázsi et al., 2008; Smollett et al., 2012a; Peterson et al., 2014; Supplementary Table 1), and then carried out similar enrichment analysis. Metabolic pathways, groups of genes in closely related functions, and transcription regulons/subnetworks with adjusted *p*-values (*p* ≤ 0.05) are considered significantly enriched. Networks were produced with Cytoscape (Shannon et al., 2003).

## Results and discussion

### Change of *M. tuberculosis* physiology in reaeration lag phase

**Figure 1:**
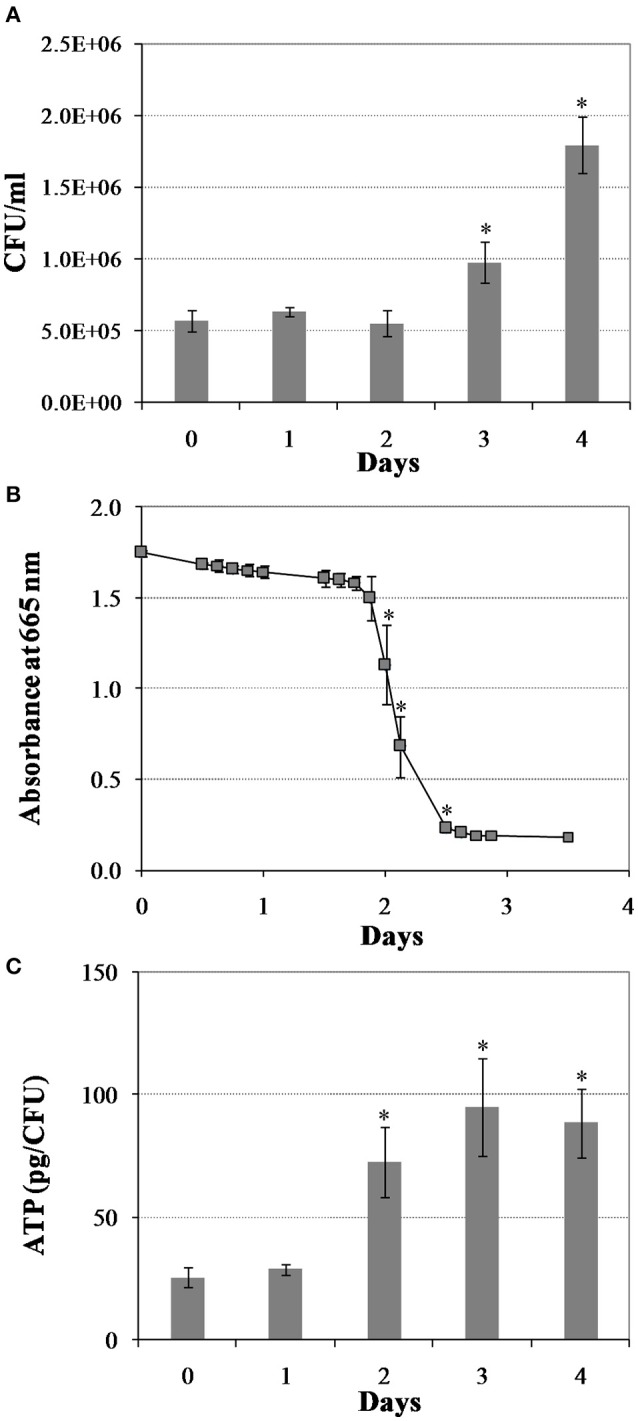
**Changes in *M. tuberculosis* physiology during reactivation from hypoxia-induced persistence**. **(A)** Colony forming units (CFU) during regrowth. Cultures were grown for 25 days in the Wayne low oxygen model, diluted in fresh media and then incubated under aerobic condition. Growth was monitored by CFU enumeration. **(B)** Oxygen utilization during regrowth. Oxygen consumption by *M. tuberculosis* was measured by methylene blue decolorization at 665 nm. **(C)** Cellular ATP levels during regrowth. Shown are averages ± *SD*'s of respective measurements from three independent cultures at each time point. ^*^indicates changes at *p* ≤ 0.05 (student's *t*-test) between measurements at corresponding time points and measurement at D0 **(A,C)**, or between measurements at corresponding time points and measurement at previous time point **(B)**.

We first created a non-replicating persistent culture by subjecting *M. tuberculosis* mid-logarithmic culture to the Wayne low oxygen model for 25 days. In this model, *M. tuberculosis* is gradually depleted of oxygen, whereby after 3 days of replication the bacilli enter into a non-replicating persistent state and they are in full anaerobiosis after 9 days (Wayne and Hayes, 1996). Next, we induced bacterial regrowth by reaeration and the addition of fresh medium. Upon reaeration, *M. tuberculosis* regrowth showed a reaeration lag phase of 2 days before growth resumed, eventually reaching the exponential phase (Figure 1A). To define the changes in *M. tuberculosis* physiology during regrowth, we measured oxygen consumption and cellular ATP levels. We found a drastic increase in cellular oxygen consumption in the reactivating bacteria starting at about day 2 of reaeration (Figure 1B). This increase in oxygen consumption was concurrent with a drastic increase in cellular ATP levels (Figure 1C), indicating an enhanced aerobic metabolism in the reactivating cells. This elevated cellular ATP production and metabolic activity prior to the increase in bacterial numbers suggests that the preparation of *M. tuberculosis* for cell division is an energy-extensive process. This conclusion is consistent with the observations in other bacteria that lag phase involves multiple metabolic processes, such as repair of macromolecule damage incurred during bacteriostatic condition (Dukan and Nyström, 1998), and synthesis of cellular components necessary for regrowth (Rolfe et al., 2012).

To decipher the molecular mechanisms leading to the physiological changes and the subsequent resumption of cell division, we characterized transcriptomic dynamics of the reactivating bacilli by RNA-Seq. Twenty-five day hypoxic cultures from multiple tubes in the Wayne model were opened, mixed with equal volume of fresh DTA medium, distributed into 25 ml tubes and cultured at 37°C under aerobic conditions with magnetic bar stirring at 450 rpm (Datta et al., 2011). At defined time points, reactivating cultures were treated and harvested for RNA extraction and sequencing. Using KEGG pathway maps, KEGG BRITE functional hierarchies (http://www.genome.jp/kegg/), and the TRN, we identified changes to many metabolic pathways, functional hierarchy groups, and transcription regulons/subnetworks in the *M. tuberculosis* reaeration lag phase (Supplementary Figures 1, 2; Supplementary Table 2). Among them, one notable observation is that as *M. tuberculosis* prepares for replicative growth it up-regulates pathways involved in the synthesis of ribosomes, ribosome-associated proteins, and amino acids, which are required for increased protein synthesis essential for the recovery of bacterial metabolic/physiological functions (Supplementary Figure 1). In agreement with the increase of bacterial aerobic metabolism (Figure 1), we observed the up-regulation of genes encoding tricarboxylic acid (TCA) cycle and oxidative phosphorylation enzymes, including NADH:quinine oxidoreductase and F-type ATPase (Supplementary Figure 1). Induction of transcriptional and translational machinery including the synthesis of ribosomes and increased aerobic energy metabolism, which was repressed during hypoxia, were also observed during reaeration response after a short-term hypoxia treatment (Sherrid et al., 2010). In addition, we observed down-regulation of many genes involved in the biosynthesis of secondary metabolites and microbial metabolism in diverse environments (Supplementary Figure 2). Based on these results, we postulate that the change of *M. tuberculosis* global gene expression program in the reaeration lag phase is a programmed process necessary for an ordered exit from persistent state. The other major observations are summarized and discussed in the following sections.

### Change of *M. tuberculosis* TRN and metabolism in the reaeration lag phase

#### Down-regulation of persistence-associated regulons/subnetworks

Establishment of *M. tuberculosis* persistence is accompanied by global gene expression changes associated with activation of stress response regulatory mechanisms (Flentie et al., 2016). Among them are the activation of two component systems, such as DosRST and MprAB (Zahrt and Deretic, 2001; Park et al., 2003; Voskuil et al., 2003; Zahrt et al., 2003; Mehra et al., 2015), and signal transduction pathways mediated by stress response alternative sigma factors such as SigE and SigH (Manganelli et al., 1999, 2001, 2002, 2004a,b; Kaushal et al., 2002). For example, induction of genes/regulons including the isocitrate lyase-encoding *icl1* and methylcitrate synthase-encoding *prpC* (*GltA1*) in the SigE subnetwork via MprA-SigE envelope-stress-signaling system is associated with bacterial metabolic and physiological transformation during entry to bacterial growth arrest (Manganelli et al., 2001; Datta et al., 2011). Similarly, the induction of ClgR regulon including the Psp system in the SigE subnetwork also presumably helps maintain membrane integrity under envelope perturbing conditions (Datta et al., 2015). It was thus expected that during the return to growth-permissive conditions the genes/regulons/subnetworks that are up-regulated during persistence would be down-regulated.

**Figure 2:**
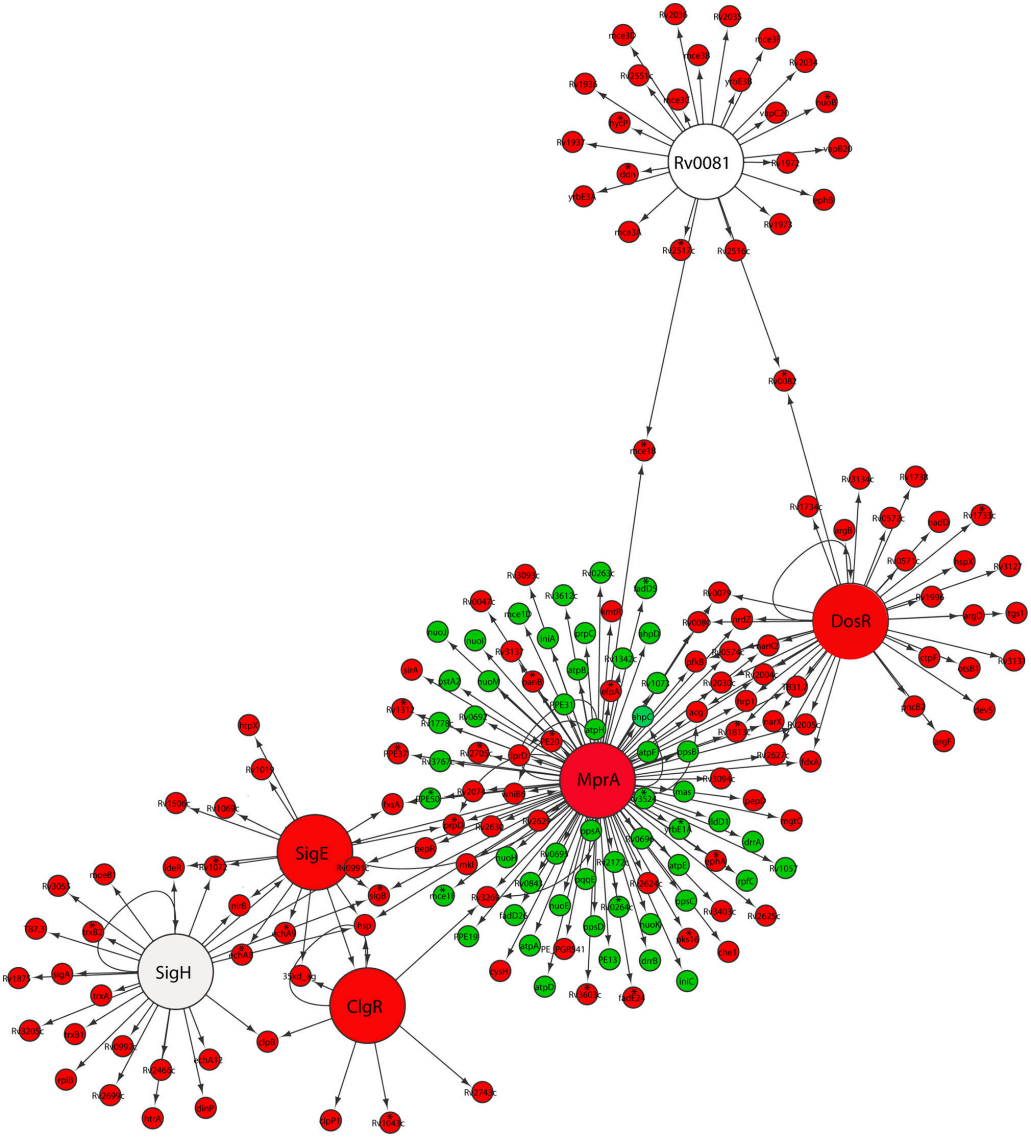
**Down-regulation of persistence-associated regulatory subnetworks in *M. tuberculosis* reaeration lag phase**. Shown are changes of gene expression in the regulons of DosR, MprA, SigE, SigH, ClgR, and Rv0081 and their connections in cultures between time points at D1 and D0, D2 and D0, or D2 and D1. Changes were identified by enrichment analysis using the transcription regulatory network. Data were derived from the RNA-Seq data of the reactivating bacilli from three independent cultures at each time point. Green color indicates up-regulation and red color denotes down-regulation. To show the connections between the regulons/subnetworks, also included are genes (marked with asterisk) whose expression showed more than 1.5-fold change but did not reach significant level (*p* ≤ 0.05) in corresponding time points.

Indeed, we found that *M. tuberculosis* reaeration lag phase was accompanied by the down-regulation of DosRST and MprAB two component signal transduction systems (Figure 2). Among them, the strong down-regulation of the DosR subnetwork, including the Rv0081 regulon, is consistent with a role in maintaining the bacilli in the persistent state (Honaker et al., 2009; Leistikow et al., 2010; He et al., 2011). Moreover, the observed down-regulation of both SigE and MprA subnetworks (Figure 2), was consistent with reports that MprA and SigE activate each other through a positive feedback loop in response to environmental stresses (He and Zahrt, 2005; He et al., 2006). We also observed down-regulation of the SigH regulon genes including *SigE* (Raman et al., 2001; Manganelli et al., 2002). Furthermore, within the SigE subnetwork, the down-regulation of *clgR* and its target genes [*Rv2743c* and *35kd_ag* (*Rv2744c*)] (Figure 2), is in agreement with the function of ClgR regulon in maintaining cell membrane/envelope functions under stress conditions and during infection of macrophages (Estorninho et al., 2010; Datta et al., 2015). This finding is in contrast to the ClgR up-regulation observed during *M. tuberculosis* regrowth induced by reaeration of a hypoxic culture (Sherrid et al., 2010). The reason for this discrepancy may be due to short duration of incubation (7 days), which does not produce fully anaerobic cultures.

The down-regulation of many genes regulated by both DosR and MprA subnetworks was in line with the notion that MprAB serves as an important link within a multifaceted response network and contributes to maintaining a balance among several systems under stress or physiological conditions (He et al., 2006; Pang et al., 2007). This is also supported by the observation that a diverse gene panel in the MprA subnetwork was up-regulated (Figure 2). Among them were genes encoding components of the NADH dehydrogenase complex (*nuo*) and ATP synthase (*atp*) involved in aerobic respiration, consistent with the increased aerobic activity and enhanced ATP production in reactivating cells (Figure 1). Another group of up-regulated genes in the MprA subnetwork were those encoding enzymes involved in the synthesis of phthiocerol dimycocerosate (PDIM), a major cell wall component of pathogenic mycobacteria. It appears that during *M. tuberculosis* reactivation the MprAB system has dual functions: the deactivation of persistence-associated mechanism as well as activation of cellular processes associated with cell regrowth. Given the fact that MprA regulates diverse gene sets in response to different stresses (He et al., 2006; Pang et al., 2007), and that many of these genes have not been studied in terms of the mechanism and specificity of their regulation by MprA, further in-depth studies are required to define whether MprA directly or indirectly regulates their expression under each specific condition.

Taken together, our data indicate that *M. tuberculosis* reaeration lag phase is marked with a deactivation of persistence-associated regulatory mechanisms, which probably constitutes an important component of the initial reactivation process. This finding is consistent with the notion that these genes play important roles during *M. tuberculosis* persistence and that their down-regulation in the reaeration lag phase is necessary to facilitate an ordered recovery of metabolic and physiological functions necessary for the eventual cell division.

#### Up-regulation of second messenger receptor protein (CRP)-mediated responses

The second messenger 3′, 5′-cyclic adenosine monophosphate (cAMP) is involved in the response to environmental cues, and plays an important role in the adaptation of *M. tuberculosis* to diverse conditions (Barba et al., 2010). The best studied member of cAMP receptor protein family of transcription regulators is CRP (Bai et al., 2011). CRP in *M. tuberculosis* regulates the expression of many genes involved in multiple processes, including persistence and/or emergence from the persistent state (Kana et al., 2008; Barba et al., 2010). A mutant strain depleted of *crp* showed impaired growth in macrophages and mice (Rickman et al., 2005). Given its functions in multiple cellular processes, CRP-mediated response was expected to be an integral part of *M. tuberculosis* reactivation process.

**Figure 3:**
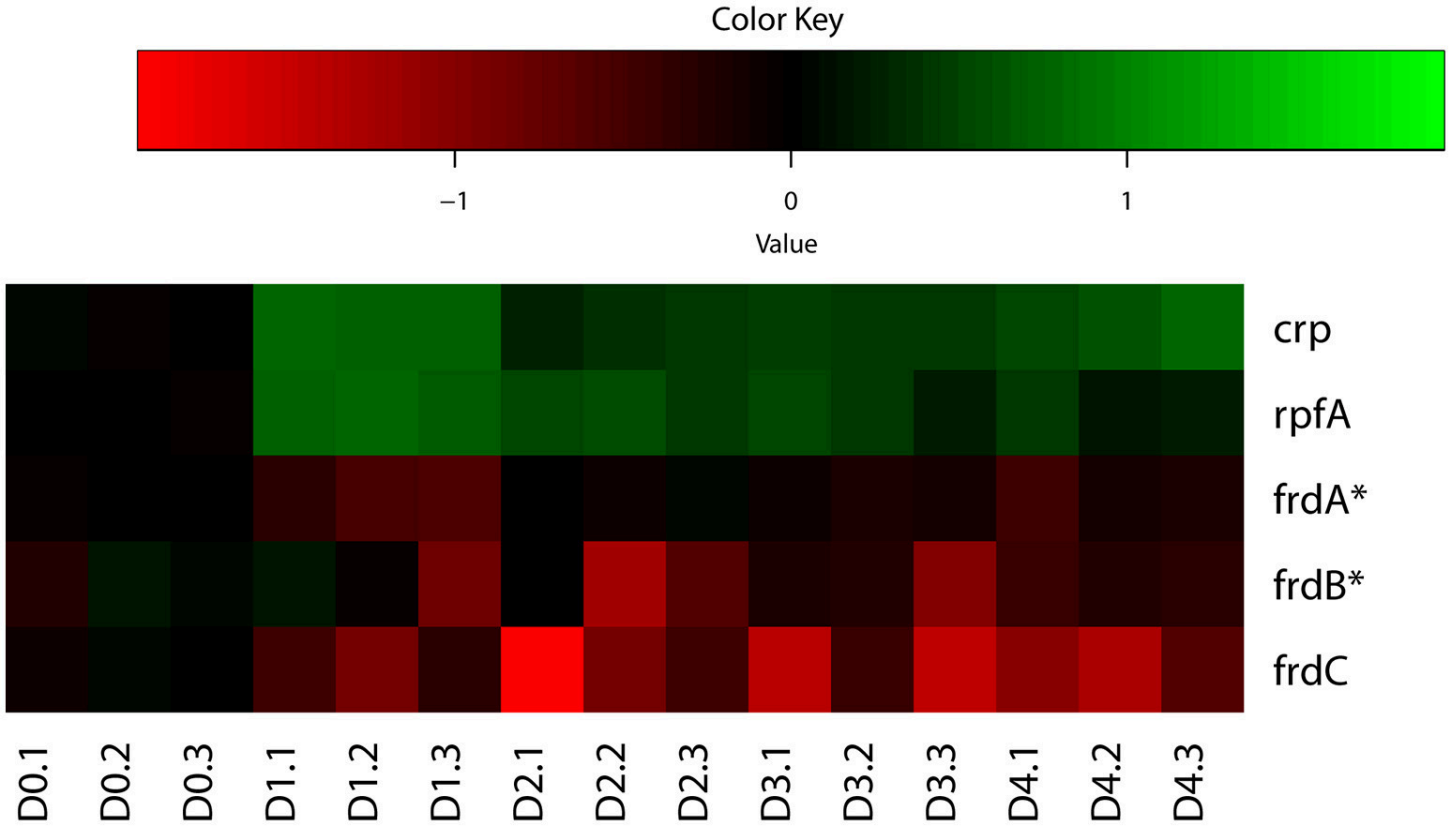
**Heatmap of representative *M. tuberculosis* CRP regulon genes during regrowth**. Changes in reactivating cultures relative to non-replicating persistent culture were identified by enrichment analysis using the expanded regulatory network. Data were derived from the RNA-Seq data of the reactivating bacilli from three independent cultures at each time point. Green color indicates up-regulation and red color denotes down-regulation. Color scale denotes log_2_ fold change in gene expression. Also included are genes (marked with asterisk) whose expression showed more than 1.5-fold change but did not reach significant level (*p* ≤ 0.05) between time points at D1 and D0, D2 and D0, or D2 and D1.

Indeed, our analysis identified both up-regulated and down-regulated genes regulated by CRP in *M. tuberculosis* reaeration lag phase (Supplementary Figure 3), consistent with the fact that CRP acts as an activator as well as a repressor of target genes depending on its binding to regions of target genes (Kahramanoglou et al., 2014). Among the various genes regulated by CRP, the up-regulation of *rpfA* (Figure 3), which encodes a resuscitation-promoting factor and was important for *M. tuberculosis* resuscitation from persistence (Rickman et al., 2005; Kana et al., 2008), underscores its critical functions during *M. tuberculosis* exit from persistence. The down-regulation of *frdC* encoding a subunit of fumarate reductase in the CRP regulon (Figure 3), is also in agreement with the role of fumarate reductase in maintaining bacterial basic physiological functions during anaerobic respiration (Watanabe et al., 2011). The down-regulation of fumarate reductase during *M. tuberculosis* reactivation thus supports a respiratory shift away from anaerobic respiration, consistent with the recovery of cellular aerobic metabolism. These data indicate that the CRP-mediated second messenger responses in *M. tuberculosis* reaeration lag phase facilitate the transitioning of bacterial metabolism and physiology to an aerobic state and the preparation for the eventual cell division.

#### Up-regulation of zinc and iron transport and mobilization

**Figure 4:**
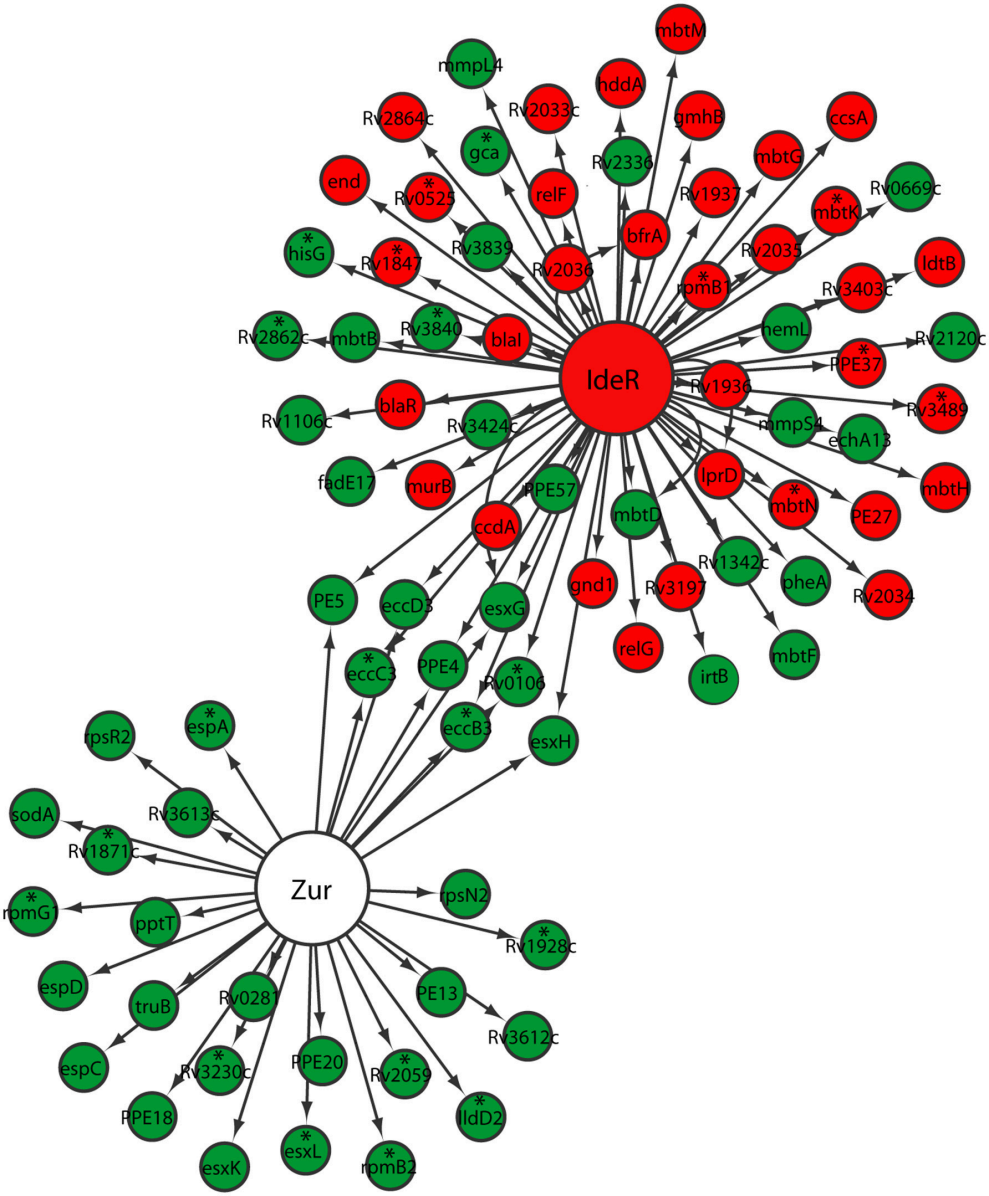
**Increased expression of genes involved in zinc and iron uptake and mobilization in *M. tuberculosis* lag phase**. Shown are changes of gene expression in the regulons of Zur and IdeR in regrowth cultures at time points between D1 and D0, D2 and D0, or D2 and D1. Changes were identified by enrichment analysis using the transcription regulatory network. Data were derived from the RNA-Seq data of the reactivating bacilli from three independent cultures at each time point. Green color indicates up-regulation and red color denotes down-regulation. To show the connections between the two regulons, also included are genes (marked with asterisk) whose expression showed more than 1.5-fold change but did not reach significant level (*p* ≤ 0.05) in corresponding time points.

Metals including zinc and iron serve as cofactors involved in many processes, including energy generation, electron transfer, DNA replication, and transcription. To compete for limited metal resources within the host while simultaneously preventing their toxicity, pathogens utilize a series of metal regulatory, acquisition, and efflux systems to maintain metal homeostasis (Becker and Skaar, 2014). During the transition between different growth states by *M. tuberculosis*, we anticipated a similar mechanism of regulation in metal transport and mobilization. Zur, encoded by *Rv2359*, is a transcription repressor that functions as a zinc uptake regulator involved in the derepression of genes involved in zinc uptake and mobilization from the storage compartment (Lucarelli et al., 2007; Maciag et al., 2007). Zur regulon genes were up-regulated in *M. tuberculosis* reaeration lag phase (Figure 4). Among them, the up-regulation of *esxG* and *esxH* in the ESX-3 secretion system, which was essential for zinc and/or iron acquisition (Serafini et al., 2009), underscores the importance of zinc and/or iron mobilization during *M. tuberculosis* reactivation. Moreover, the up-regulation of genes (*rpsR2* and *rpsN2)* encoding ribosomal proteins may be associated with zinc mobilization during *M. tuberculosis* regrowth. Products of *rpsR2* and *rpsN2* have paralogs unable to bind zinc (Maciag et al., 2007), their up-regulation during *M. tuberculosis* regrowth thus replaces the zinc-associated paralogs to facilitate zinc remobilization from storage. We also observed a trend of induction (not significantly) of genes encoding other proteins possibly involved in zinc uptake and transport. For example, the product of *Rv0106* is similar to a zinc transporter in *Bacillus subtilis* (Gaballa and Helmann, 1998), and the product of *Rv2059* belongs to a superfamily of proteins functioning as initial receptors in ABC transport of Zn^2+^ and Mn^2+^ in many eubacterial species (Lee et al., 2002; Figure 4). Thus, *M. tuberculosis* reaeration lag phase is marked with enhanced zinc uptake and mobilization, which is expected to facilitate the recovery of bacterial cellular and metabolic functions.

While also regulated by the ESX-3 secretion system in the Zur regulon, iron homeostasis in mycobacteria is mainly regulated by IdeR, an iron-dependent repressor and activator (Gold et al., 2001). Since iron serves as cofactor for proteins/enzymes with various functions including those involved in aerobic respiration (Pantopoulos et al., 2012), we expected increased iron uptake and remobilization as *M. tuberculosis* transitioned from an anaerobic to aerobic environment. Indeed, *irtB*, which encodes a component of ABC transporters for Fe-carboxymycobactin (Rodriguez and Smith, 2006), was up-regulated (Figure 4). Consistent with elevated iron mobilization, *bfrA*, involved in the synthesis of bacterioferritin, a principal iron storage molecule (Gold et al., 2001), was down-regulated. However, genes in the two gene clusters involved in the biosynthesis of siderophores (Quadri et al., 1998; Krithika et al., 2006), displayed divergent expression profiles. For example, *mbtB, mbtD*, and *mbtF* in the *mbt1* cluster (*mbtA-J*; Quadri et al., 1998), were induced, while *mbtG* in the *mbt1* cluster and *mbtM* in the *mbt2* cluster (*mbtK-N*; Krithika et al., 2006), were down-regulated (Figure 4). This observation suggests that siderophore biosynthesis is not necessarily associated with *M. tuberculosis* reactivation, perhaps reflecting a lack of iron deficiency in the regrowth environment. This is consistent with the notion that iron transporters are required for iron mobilization under conditions with low iron, but siderophore biosynthesis is only active during iron depletion (De Voss et al., 2000). Our data thus indicate that *M. tuberculosis* reaeration lag phase is associated with increased iron acquisition by active transport and remobilization of storage iron.

#### Up-regulation of DNA repair and recombination

As an intracellular pathogen, *M. tuberculosis* encounters a variety of DNA-damaging conditions during infection, primarily from host-generated antimicrobial reactive oxygen and nitrogen species (Nathan and Shiloh, 2000). DNA repair is critical for slowly dividing cells, where unrepaired damage tends to accumulate during non-replicating persistence. DNA damage response pathways in *M. tuberculosis* include the RecA/LexA-dependent SOS response and RecA/LexA-independent pathways (Davis et al., 2002b; Rand et al., 2003; Dos Vultos et al., 2009; Smollett et al., 2012b). Given the high possibility of DNA damage occurring during persistence as well as reaeration-induced regrowth, we expected DNA repair processes to be active in *M. tuberculosis* reaeration lag phase.

**Figure 5:**
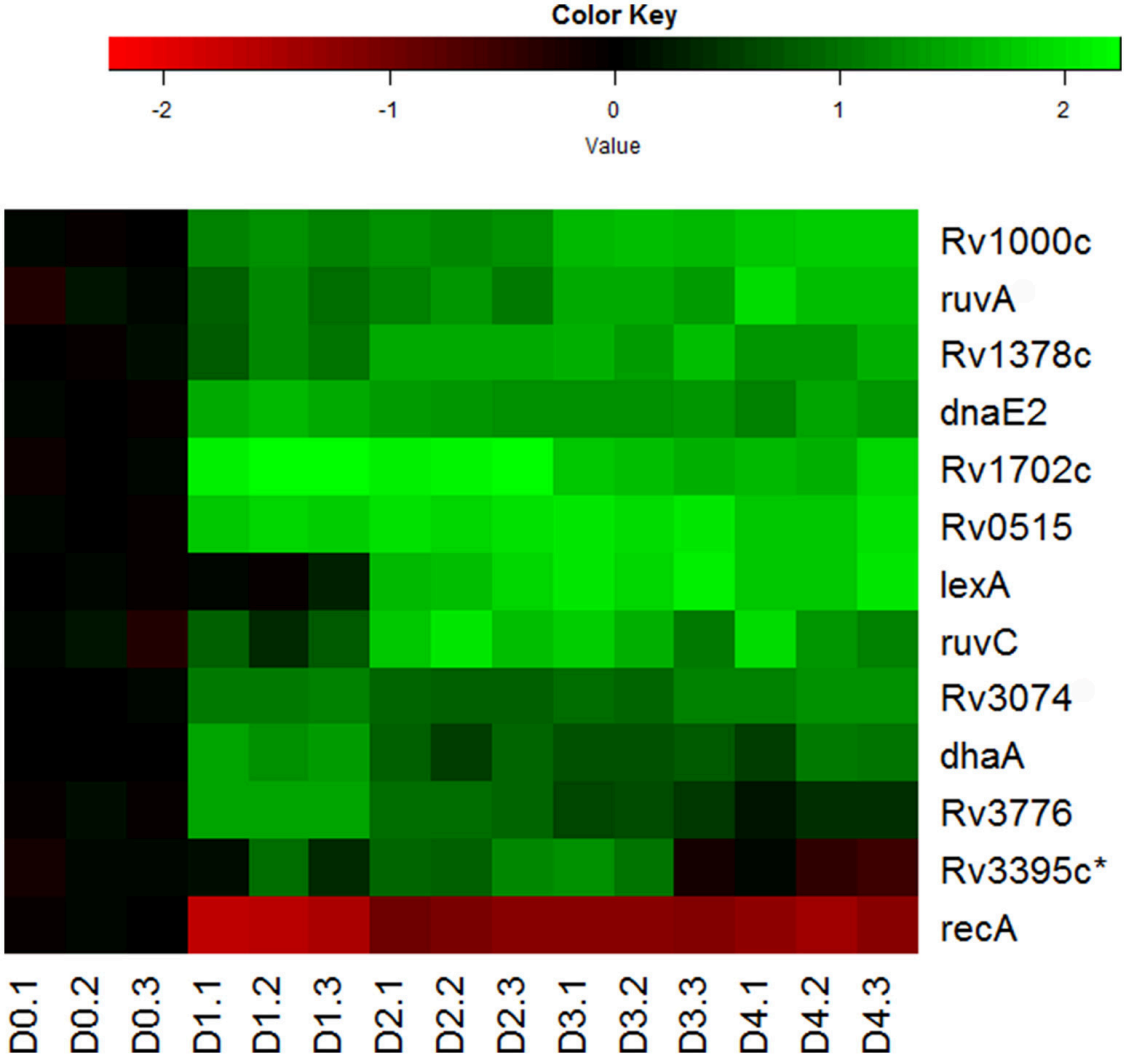
**Increased expression of genes involved in DNA repair and recombination during *M. tuberculosis* regrowth**. Shown are the expression changes of genes in the LexA regulon during regrowth. Changes relative to non-replicating persistent culture were identified by enrichment analysis using the transcription regulatory network. Data were derived from the RNA-Seq data of the reactivating bacilli from three independent cultures at each time point. Green color indicates up-regulation and red color denotes down-regulation. Color scale denotes log_2_ fold change in gene expression. Asterisk indicates that the change of gene expression was more than 1.5-fold change but did not reach significant level (*p* ≤ 0.5) between time points at D1 and D0, D2 and D0, or D2 and D1.

Indeed, we observed the up-regulation of most genes associated with LexA-mediated SOS response (Smollett et al., 2012b; Figure 5). Among them, *ruvA, ruvC*, and *dnaE2* are directly associated with DNA repair (Davis et al., 2002a; Boshoff et al., 2003), while other genes in this regulon, such as *Rv0515, Rv1702c, Rv3074*, and *Rv3776*, are probable members of the *M. tuberculosis* 13E12 repeat family, which has characteristics of mobile elements that become active when DNA damage occurs (Davis et al., 2002a). Not so surprisingly, *recA* expression in the regulon was found to be down-regulated (Figure 5). Although RecA, in conjunction with repressor protein LexA, controls the expression of this set of DNA repair genes, its down-regulation is consistent with the findings that *recA* is also regulated by LexA-independent mechanisms (Brooks et al., 2001; Davis et al., 2002b; Smollett et al., 2012b). The LexA independent promoter of *recA* was shown to be regulated by ClpR protein-like regulator, encoded by *clgR* in *M. tuberculosis* (Gamulin et al., 2004; Wang et al., 2012). In addition, we detected the up-regulation in the reaeration lag phase of many other DNA repair and recombination genes, such as *uvrA, recN*, and *uvrD1* (Movahedzadeh et al., 1997; Brooks et al., 2001; Boshoff et al., 2003; Rand et al., 2003; Supplementary Table 2). These data are in agreement with other reports that many DNA repair genes in *M. tuberculosis* are induced independently of RecA (Boshoff et al., 2003; Brooks et al., 2001; Movahedzadeh et al., 1997; Rand et al., 2003). Taken together, our observations suggest that DNA repair and recombination is an integral part of *M. tuberculosis* reactivation program in the reaeration lag phase.

#### Up-regulation of the synthesis of major cell wall lipids

The *M. tuberculosis* envelope is composed of a cell wall core and multiple noncovalently attached capsular lipids. The cell wall core includes peptidoglycans covalently attached to arabinogalactans, which are in turn attached to mycolic acids; the noncovalently associated components consist of multimethyl-branched lipids including sulfolipids and PDIMs, which are produced by the combined action of fatty acid synthases and polyketide synthases (Daffé and Laneelle, 1988). Since these complex lipids comprise 40–60% of cellular dry weight (Goren, 1972), their synthesis was expected to constitute a rate-limiting process during *M. tuberculosis* reactivation and to serve as an energy and carbon flux sink. Thus, it was expected that an increased expression of genes involved in their synthesis in reaeration lag phase would be a necessary step in the preparation for cell division.

**Figure 6:**
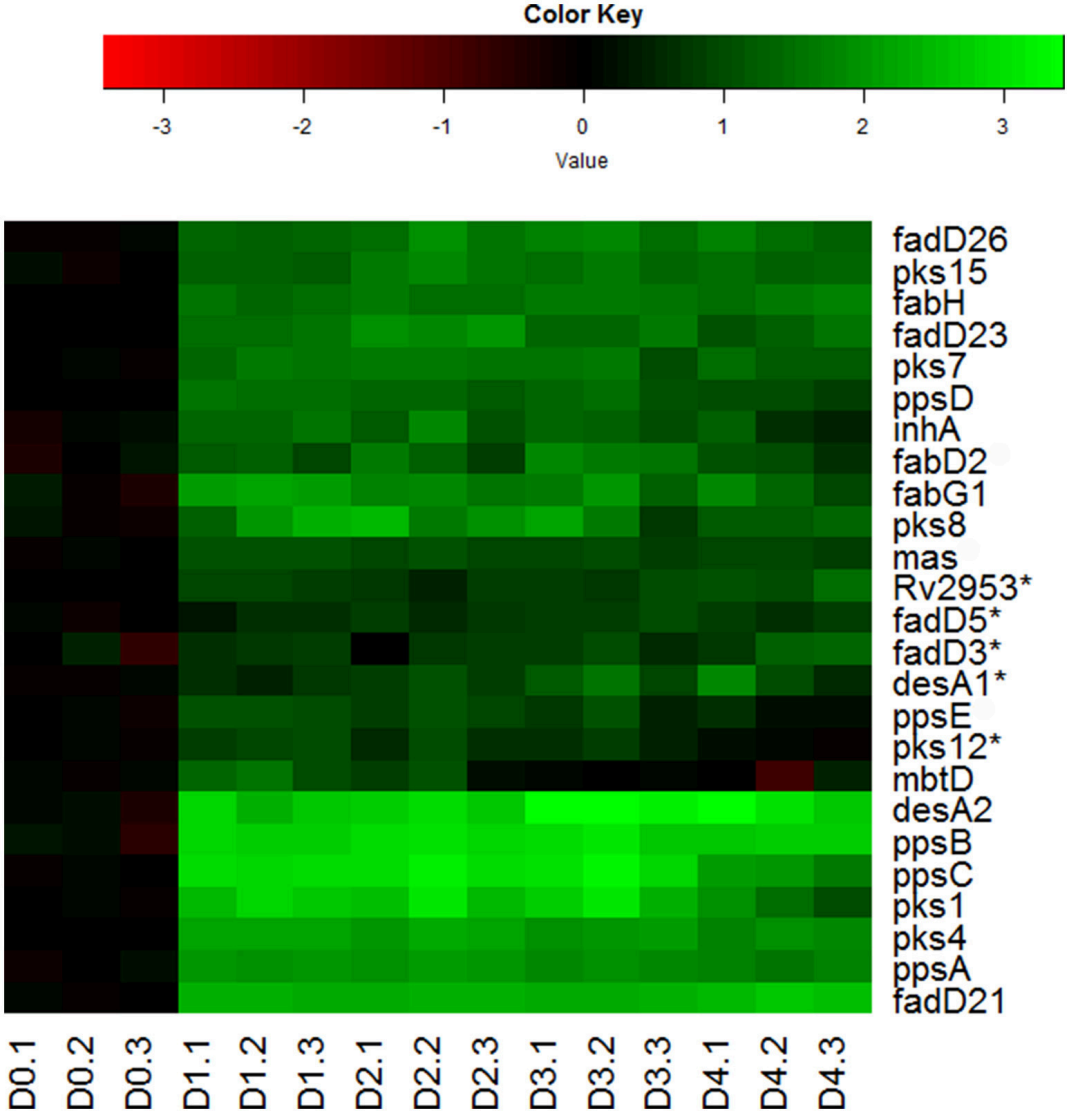
**Increased expression of genes involved in synthesis of major cell wall components during *M. tuberculosis* regrowth**. Changes of genes involved in major cell wall synthesis during *M. tuberculosis* regrowth relative to non-replicating persistent culture were identified by enrichment using the KEGG pathway and KEGG BRITE hierarchies. Data were derived from the RNA-Seq data of the reactivating bacilli from three independent cultures at each time point. Green color indicates up-regulation and red color denotes down-regulation. Color scale denotes log_2_ fold change in gene expression. Also included are genes (marked with asterisk) whose expression showed more than 1.5-fold change but did not reach significant level (*p* ≤ 0.5) between time points at D1 and D0, D2 and D0, or D2 and D1.

Indeed, we observed the up-regulation of many genes encoding key enzymes involved in the synthesis of mycolic acids, PDIM and sulfolipids (Figure 6). During mycolic acid synthesis, the product of *fabH* serves as a critical link between the two FAS systems (FAS-I and II) of mycolic acid synthesis by catalyzing the condensation between FAS-I acyl-CoA primers and malonyl-AcpM, and funneling precursors to FAS-II (Choi et al., 2000). Up-regulation of *fabH* in reaeration lag phase (Figure 6), may thus play an important role in directing the carbon flux from FAS-I toward mycolic acid synthesis. In line with this notion, genes encoding key enzymes of FAS-II, such as *fabG1* and *inhA*, were also induced in the reaeration lag phase (Figure 6), indicating that there is a concerted carbon flow toward the formation of mycolic acid. Also up-regulated were a group of genes (*mas, fadD26*, and *ppsA-E)* involved in the synthesis and translocation of PDIM, a major component of noncovalently attached lipids of the *M. tuberculosis* cell wall. Up-regulation of PDIM synthesis was also found during the reaeration response after a short-term hypoxia treatment (Sherrid et al., 2010). In addition, several *pks* genes (*pks1, psk4, pks7, pks8*, and *pks15*) encoding polyketide synthases that participate in the synthesis of complex lipids including sulfolipids were induced (Figure 6). Together, these findings point to enhanced synthesis of major cell wall lipids in *M. tuberculosis* during the emergence from persistence and preparation for cell division.

#### Down-regulation of lipid uptake and catabolism

*M. tuberculosis* utilizes diverse lipids as major carbon and energy source during infection. Upon entry to the persistent state, *M. tuberculosis* carbon metabolism was associated with increased fatty acid scavenging, oxidation, and assimilation of fatty acid oxidation products via the induction of glyoxylate shunt (Shi et al., 2010). Additionally, cholesterol uptake and utilization were shown to be required for *M. tuberculosis* survival during persistent stage of the infection (Pandey and Sassetti, 2008). In contrast to the up-regulation of lipid uptake and assimilation upon entry into persistence, during reactivation we observed down-regulation of multiple pathways involved in the uptake of lipids and their catabolism. First, fatty acid beta-oxidation and degradation pathways were down-regulated in the reaeration lag phase (Supplementary Figure 2). Second, we detected the down-regulation of genes regulated by KstR (Supplementary Figure 4), a TetR-like transcriptional repressor that controls the expression of a cluster of mycobacterial genes involved in the lipid degradation, especially in cholesterol catabolism (Kendall et al., 2007; Van der Geize et al., 2007). Third, we identified the down-regulation of Mce transport systems (Mce1-4; Supplementary Figure 5), which encode putative ABC transporters involved in diverse lipid transportation across the cell wall (Casali and Riley, 2007; Pandey and Sassetti, 2008). Fourth, consistent with decreased lipid uptake and catabolism, we observed down-regulation of genes involved in glyoxylate and dicarboxylate metabolism (Supplementary Figure 2), a canonical pathway for lipid utilization.

Given the fact that there was no cholesterol in the regrowth media, the observed down-regulation of genes involved in cholesterol catabolism may reflect a hard-wired gene expression program that is associated with change of *M. tuberculosis* growth state rather than with the nutrition status. This notion is supported by similar finding that that up-regulation of the glyoxylate shunt is not necessarily associated with its canonical role of lipid utilization but rather with a non-canonical role in the metabolic reprogramming that occurs during a change in *M. tuberculosis* growth states (Shi et al., 2010; Eoh and Rhee, 2013).

### Diminished ability of *M. tuberculosis* mutants to recover from persistence

**Figure 7:**
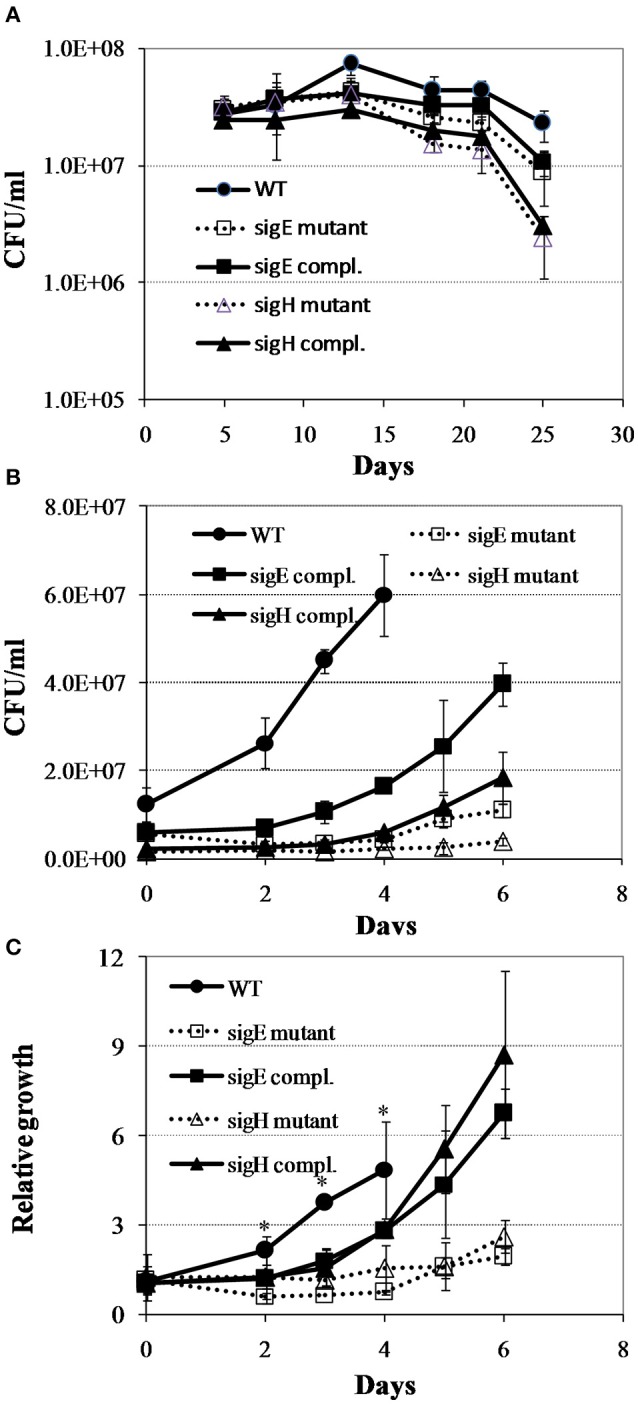
**Regrowth dynamics of *M. tuberculosis sigE* and *sigH* mutants during exit from hypoxia-induced persistence**. **(A)** Growth curves in the Wayne low oxygen model. **(B)** Regrowth curves during reactivation from non-replicating persistent state. **(C)** Relative regrowth during recovery from non-replicating persistent state. Cultures were grown for 25 days in the Wayne low oxygen model, diluted in fresh media, and then grown under aerobic condition. Growth was monitored by CFU enumeration. The initial cell density was adjusted to an arbitrary unit of 1.0 and measurements at each subsequent time point were reported as relative to this initial value. Shown are averages ± *SD*'s of measurements from three independent cultures at each time point. ^*^indicates significant changes at *p* ≤ 0.05 (student's *t*-test) between the mutant strains and WT at identical time points **(C)**.

To test the notion that the global gene expression program in *M. tuberculosis* reaeration lag phase constitutes the molecular basis necessary for a normal recovery of bacterial metabolic and physiological functions leading to the eventual cell replication after prolonged hypoxia, we characterized the regrowth dynamics of *M. tuberculosis* mutants lacking genes encoding SigE or SigH. These sigma factors are known to regulate many genes involved in stress adaptations that are up-regulated during persistence and down-regulated in the reaeration lag phase. The *sigH* mutant and its complemented strain showed decrease in survival at the final time point in comparison to the WT strain (Figure 7A). During regrowth, a prolonged reaeration lag phase and significantly slower recovery were observed in both mutant strains compared to the WT and complemented strains (Figures 7B,C). This observation suggests that a coordination of global gene expression program including the down-regulation of persistence-associated regulons/subnetworks in the *M. tuberculosis* reaeration lag phase is necessary for an ordered transition to replicative growth.

## Conclusions

In this study we dissected the molecular mechanisms of *M. tuberculosis* reactivation from prolonged hypoxia-induced non-replicating persistence by characterizing bacterial physiology and transcriptomic dynamics. Our analysis uncovered an initial transcriptional reactivation program in *M. tuberculosis* reaeration lag phase, which includes a coordinated change of many regulatory mechanisms and metabolic pathways and is essential for a programmed exit from non-replicating persistence. In particular, the identification of up-regulated metabolic pathways and transcription regulons/subnetworks, including the uptake and remobilization of iron and zinc, DNA repair and recombination, and synthesis of major cell wall components, highlights potential therapeutic metabolic intervention checkpoints to prevent *M. tuberculosis* from reactivation. Considering the fact that these observations are derived from reactivation of persistent cultures from a well-controlled environment, and that the reactivation of a human latent infection may come from changes in a more diverse environment, further studies of *M. tuberculosis* gene expression during reactivation from *in vivo* infection are needed to substantiate our findings.

## Author contributions

PD analyzed the RNA-Seq data and participated in writing the manuscript. CS designed and carried out experiments, analyzed data, and wrote the manuscript; and LS designed and performed experiments, analyzed and interpreted data, and wrote the manuscript.

### Conflict of interest statement

The authors declare that the research was conducted in the absence of any commercial or financial relationships that could be construed as a potential conflict of interest.

## Supplementary material

Supplementary Table 1 **Transcription factors and their target genes in *M. tuberculosis***.

Supplementary Table 2 **Changes in the expression of *M. tuberculosis* genes in the reaeration lag phase**.

Supplementary Figure 1 **Heatmap of up-regulated metabolic pathways and transcription regulons in *M. tuberculosis* reaeration lag phase**. Significant changes in reactivating cultures between D1 and D0, D2 and D0, or D2 and D1 were identified by enrichment analysis using the KEGG metabolism map, KEGG BRITE hierarchies, and the transcription regulatory network. Color key denotes levels of significance (adjusted *p* values): gray (≤ 0.05); light blue (10^−3^ – 0.05), blue (10^−5^ – 10^−3^); and dark blue (≤ 10^−5^).

Supplementary Figure 2 **Heatmap of down-regulated metabolic pathways and transcription regulons in *M. tuberculosis* reaeration lag phase**. Significant changes in reactivating cultures between D1 and D0, D2 and D0, or D2 and D1 were identified by enrichment analysis using the KEGG metabolism map, KEGG BRITE hierarchies, and the transcription regulatory network. Color key denotes levels of significance (adjusted *p* values): gray (≤ 0.05); light blue (10^−3^ – 0.05), blue (10^−5^ – 10^−3^); and dark blue (≤ 10^−5^).

Supplementary Figure 3 **Heatmap of *M. tuberculosis* CRP regulon genes during regrowth**. Changes in reactivating cultures relative to non-replicating persistent culture were identified by enrichment analysis using the expanded regulatory network. Data were derived from the RNA-Seq data of the reactivating bacilli from three independent cultures at each time point. Green color indicates up-regulation and red color denotes down-regulation. Color scale denotes log_2_ fold change in gene expression.

Supplementary Figure 4 **Down-regulation of KstR regulon genes during *M. tuberculosis* regrowth**. Changes in reactivating cultures relative to non-growing persistent culture were identified by enrichment analysis using the transcription regulatory network. Data were derived from the RNA-Seq data of the reactivating bacilli from three independent cultures at each time point. Green color indicates up-regulation and red color denotes down-regulation. Color scale denotes log_2_ fold change in gene expression. Also included in the regulon are genes (marked with asterisk) whose expression showed more than 1.5-fold change but did not reach significant level (*p* ≤ 0.05) between time points at D1 and D0, D2 and D0, or D2 and D1.

Supplementary Figure 5 **Down-regulation of gene expression in Mce transport systems during *M. tuberculosis* regrowth**. Changes in reactivating cultures relative to non-growing persistent culture were identified by enrichment analysis using KEGG metabolic map and KEGG BRITE hierarchies. Data were derived from the RNA-Seq data of the reactivating bacilli from three independent cultures at each time point. Green color indicates up-regulation and red color denotes down-regulation. Color scale denotes log_2_ fold change in gene expression. Also included in the regulon are genes (marked with asterisk) whose expression showed more than 1.5-fold change but did not reach significant level (*p* ≤ 0.05) between time points at D1 and D0, D2 and D0, or D2 and D1.
