# Supplementary figures and images for: Transcriptional and Physiological Changes during Mycobacterium tuberculosis Reactivation from Non-replicating Persistence
Source: Front Microbiol. 2016 Aug 31;7:1346. doi: 10.3389/fmicb.2016.01346 (PMC5005354; doi:10.3389/fmicb.2016.01346)

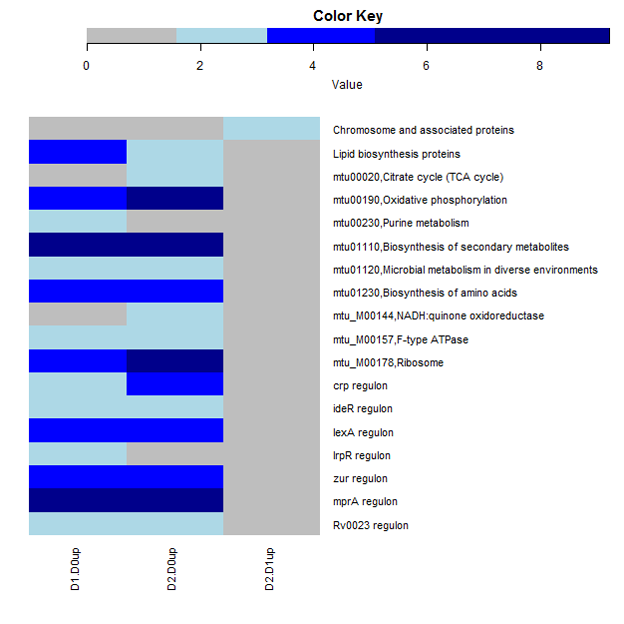

Supplement: Supplementary Figure 1 — Heatmap of up-regulated metabolic pathways and transcription regulons in M. tuberculosis reaeration lag phase. Significant changes in reactivating cultures between D1 and D0, D2 and D0, or D2 and D1 were identified by enrichment analysis using the KEGG metabolism map, KEGG BRITE hierarchies, and the transcription regulatory network. Color key denotes levels of significance (adjusted p values): gray (≤ 0.05); light blue (10−3 – 0.05), blue (10−5 – 10−3); and dark blue (≤ 10−5). [file Image1.TIFF]

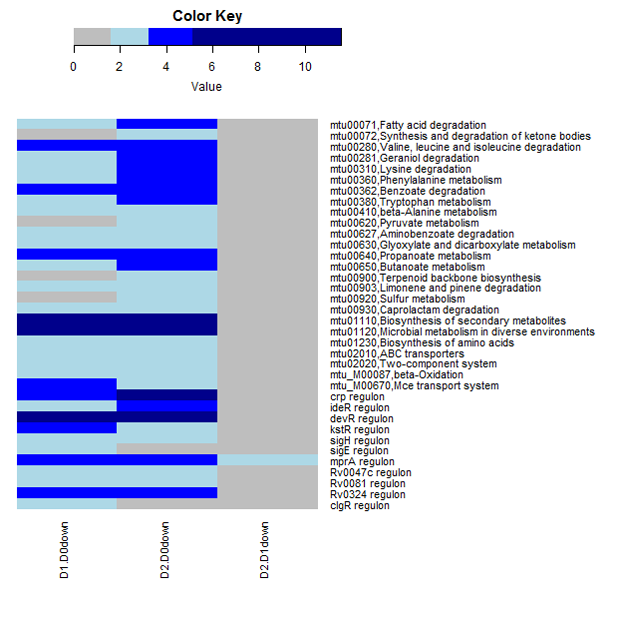

Supplement: Supplementary Figure 2 — Heatmap of down-regulated metabolic pathways and transcription regulons in M. tuberculosis reaeration lag phase. Significant changes in reactivating cultures between D1 and D0, D2 and D0, or D2 and D1 were identified by enrichment analysis using the KEGG metabolism map, KEGG BRITE hierarchies, and the transcription regulatory network. Color key denotes levels of significance (adjusted p values): gray (≤ 0.05); light blue (10−3 – 0.05), blue (10−5 – 10−3); and dark blue (≤ 10−5). [file Image2.TIFF]

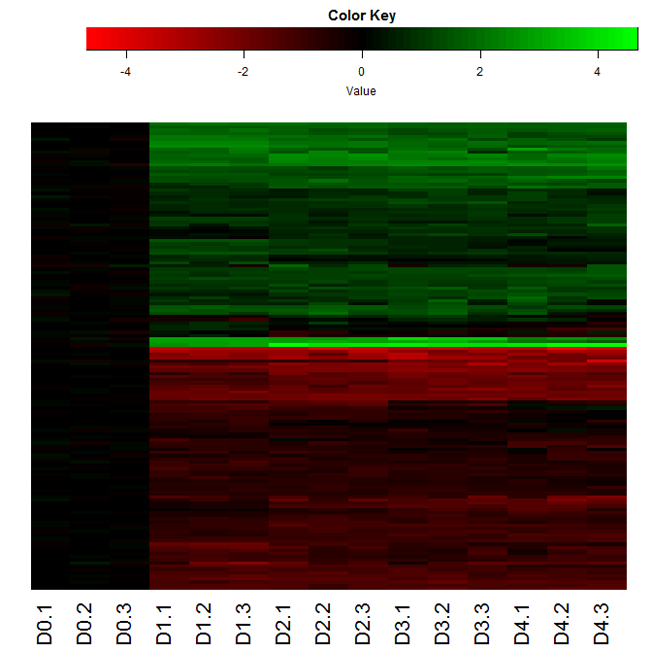

Supplement: Supplementary Figure 3 — Heatmap of M. tuberculosis CRP regulon genes during regrowth. Changes in reactivating cultures relative to non-replicating persistent culture were identified by enrichment analysis using the expanded regulatory network. Data were derived from the RNA-Seq data of the reactivating bacilli from three independent cultures at each time point. Green color indicates up-regulation and red color denotes down-regulation. Color scale denotes log2 fold change in gene expression. [file Image3.TIFF]

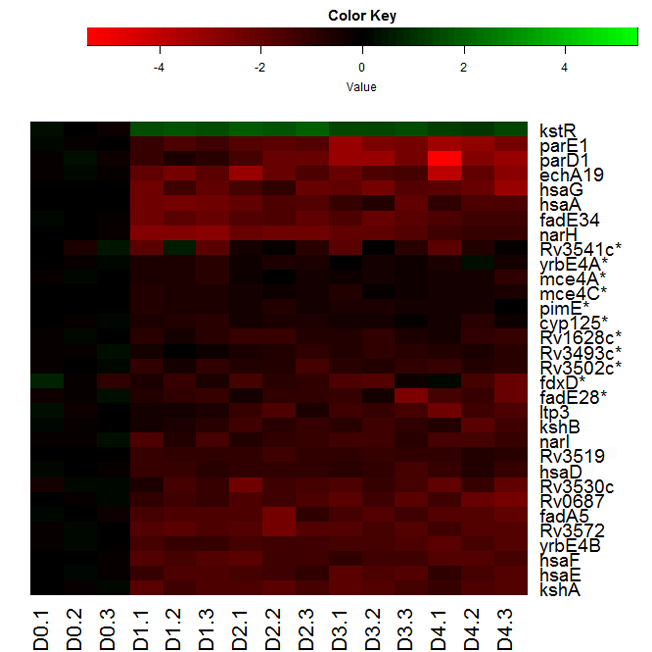

Supplement: Supplementary Figure 4 — Down-regulation of KstR regulon genes during M. tuberculosis regrowth. Changes in reactivating cultures relative to non-growing persistent culture were identified by enrichment analysis using the transcription regulatory network. Data were derived from the RNA-Seq data of the reactivating bacilli from three independent cultures at each time point. Green color indicates up-regulation and red color denotes down-regulation. Color scale denotes log2 fold change in gene expression. Also included in the regulon are genes (marked with asterisk) whose expression showed more than 1.5-fold change but did not reach significant level (p ≤ 0.05) between time points at D1 and D0, D2 and D0, or D2 and D1. [file Image4.TIF]

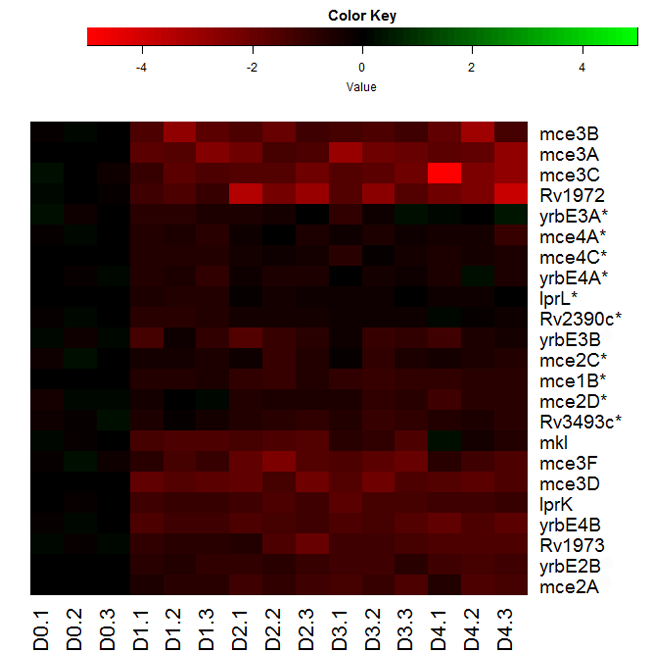

Supplement: Supplementary Figure 5 — Down-regulation of gene expression in Mce transport systems during M. tuberculosis regrowth. Changes in reactivating cultures relative to non-growing persistent culture were identified by enrichment analysis using KEGG metabolic map and KEGG BRITE hierarchies. Data were derived from the RNA-Seq data of the reactivating bacilli from three independent cultures at each time point. Green color indicates up-regulation and red color denotes down-regulation. Color scale denotes log2 fold change in gene expression. Also included in the regulon are genes (marked with asterisk) whose expression showed more than 1.5-fold change but did not reach significant level (p ≤ 0.05) between time points at D1 and D0, D2 and D0, or D2 and D1. [file Image5.TIF]
